# Atomically Dispersed Electrocatalysts for Oxygen Reduction Reaction: Understanding the Synthetic Processes for Tuning Structure, Surface Chemistry, and Formation of Different Active Sites

**DOI:** 10.1021/acsaem.5c00687

**Published:** 2025-05-28

**Authors:** Mohsin Muhyuddin, Enrico Berretti, Camille Roiron, Alessandro Lavacchi, Iryna V. Zenyuk, Plamen Atanassov, Carlo Santoro

**Affiliations:** † Electrocatalysis and Bioelectrocatalysis Laboratory, Department of Materials Science, 9305 University of Milano-Bicocca, U5, Via Cozzi 5, 20125, Milano, Italy; ‡ Istituto di Chimica Dei Composti OrganoMetallici (ICCOM), Consiglio Nazionale Delle Ricerche (CNR), Via Madonna Del Piano 10, 50019 Sesto Fiorentino, Firenze, Italy; § Department of Chemical and Biomolecular Engineering, University of California, Irvine, California 92697, United States; ∥ Department of Materials Science & Engineering, 8788 University of California Irvine, Irvine, California 92617, United States

**Keywords:** Fe-N_*x*_-C electrocatalysts, oxygen reduction reaction, PGM-free, synthetic procedures, in situ pyrolysis

## Abstract

The interest in atomically dispersed transition metal (TM-N_*x*_-C) electrocatalysts for a plethora of electrochemical reactions has risen dramatically due to their superior selective activities and operational durability. Concerning the oxygen reduction reaction (ORR), diverse simple and complex synthetic routes are used to synthesize TM-N_*x*_-C. However, each known route exploits a pyrolysis step to (i) stabilize the TM-N_*x*_ over the carbon support, (ii) create active sites, and (iii) enhance graphitization. Commonly, the synthetic routes also involve a postpyrolytic treatment to enhance atomic level homogenization/distribution of the active moieties and remove undesired active sites such as nanoparticles and oxides. Synthetic processes are discussed and the reaction mechanisms are highlighted. Recently, in situ characterization methods and techniques have unraveled the evolution, growth, and then transformation of active site structure and morphological attributes during the pyrolytic process. In this review, the synergistic effects of surface chemistry and morphology of these electrocatalysts are reported and discussed.

## Graphical abstract

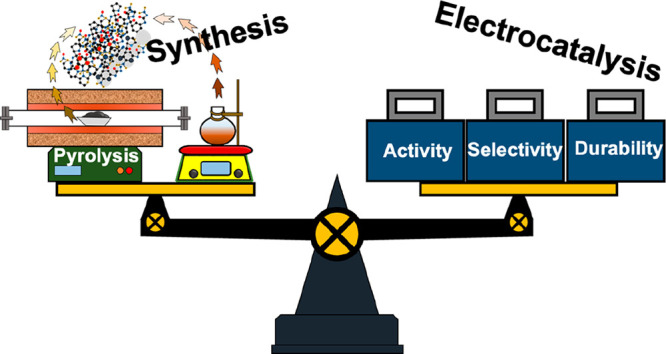

## Introduction

Many electrochemical reactions such as the oxygen reduction reaction (ORR), carbon dioxide reduction reaction (CO_2_RR), and nitrate reduction reaction (NRR) are critical for decarbonization technologies, as they rely on these processes to generate useful products.

Concentrating the attention on platinum group metal (PGM)-based electrocatalysts that are expensive, rare, and geographically located in specific regions, a need to find efficient alternatives that are low-cost and readily available is imperative. Therefore, the alternatives were searched by the direct replica of naturally existing classes of electrocatalysts based on enzymes in which heterocyclic macrocycle complex organic compounds act as the active sites.

In the case of the ORR, early bioinspired electrocatalysts are reflected in the findings regarding active sites and cofactors of enzymes involved in charge transfer (cytochromes), oxygen transfer (myoglobin and hemoglobin), and respiratory chain (cytochrome C oxidase). All of those involve using earth-abundant transition metals such as Fe, Cu, and Mn in various coordination environments. In many species of protozoa, plants, and fungi, as well as a great domain of the animal kingdom, the enzymatic ORR is catalyzed by Cu-containing/dependent enzymes, often associated with complex, multidomain protein structures. In the remaining domains of the animal kingdom (including mammals) as well as several types of plants, Fe-containing charge-transfer, oxygen-transport, and ORR-catalyzing enzymes dominate.^1^ Bioinspired approaches have been focusing initially on the design of ORR catalysts after heme-containing protein structures and initially involved porphyrins or phthalocyanines, planar molecular complexes (chelates), in which a transition metal is coordinated with four pyrrolic nitrogens. One can mark 1964 as the year of origin for this approach, as it is then for the first time that Raymond Jasinski reported the on the use of Co-phthalocyanine as an ORR electrocatalyst, even claiming its utility in fuel cells.^2^

Since then, a lot of effort has been devoted in this direction by studying the direct replica of what is occurring in nature, focusing on this class of electrocatalysts resembling the structures of metallo-porphyrins and also other similar structures such as phthalocyanine and other atomically dispersed species containing azamacrocycles.^3^ Many initial studies were carried out by anchoring these metal–nitrogen macro complexes over a conductive carbon support. However, three main problems were observed over time: (i) atomically dispersed transition metals (TM) azamacrocycle-like structures are not stable in the long run and degrade very easily, (ii) these active sites produce an important quantity of intermediates (peroxide), therefore leading to an incomplete ORR, and (iii) the cost of these heterocyclic macrocycle complex organic compounds is not necessarily low.

A fundamental and important breakthrough was brought to light in 2002 when it was shown that the coordination of iron in a pyridinic nitrogen environment instead of a pyrrolic nitrogen environment is more efficient leading to an enhanced electrocatalysis.^4^ Passing then from a biomimetic approach to a bioinspired design using pyrolytic processes, the synthesized electrocatalysts met the requirement of technology by possessing low overpotentials and producing low or negligible intermediate and high current density.

This short review briefly describes the different categories of TM-N_*x*_-C based on different synthetic routes to fabricate them. Moreover, the different active sites containing the atomically dispersed TM coordinated with nitrogen (TM-N_*x*_), TM nanoparticles or oxides, or TM-free active sites are enumerated and described, and the reaction mechanisms during the ORR of each of these active sites are described in detail. The great majority of the PGM-free TM-N_*x*_-C electrocatalysts are synthesized using high-temperature processes, named pyrolytic processes, and therefore, surface chemistry and morphology are usually investigated initially (before pyrolysis) and at the end (after pyrolysis). The main focus of this perspective is related to the great effort that has been invested in the past few years (since 2019), into understanding the transformations occurring during the pyrolytic processes. The review puts its attention on the formations of the desired active sites during pyrolysis by using a plethora of in situ microscopic and spectroscopic tools, often supported by a light synchrotron. The development of novel tools and the application of in situ microscopy and spectroscopic techniques described in this review allowed the tracking of the active site formation and the variations of morphology and unraveling of what is occurring during pyrolysis to the surface chemistry and morphology.

## Synthetic Methods Using Pyrolytic Processes

Atomically dispersed electrocatalysts of the family TM-N_*x*_-C with *x* = 2, 3, 4 are composed of over 90% of a carbon structure that is the backbone of the material guaranteeing electrical conductivity. The remaining part is composed of nitrogen alone as nitrogen functional groups or coordinated with the TM in the TM-N_*x*_-C with *x* = 2, 3, 4.^5–7^

The synthetic pathways to produce these PGM-free electrocatalysts are different, however, and can be classified into four main categories, as shown in Figure 1.

**Figure 1:**
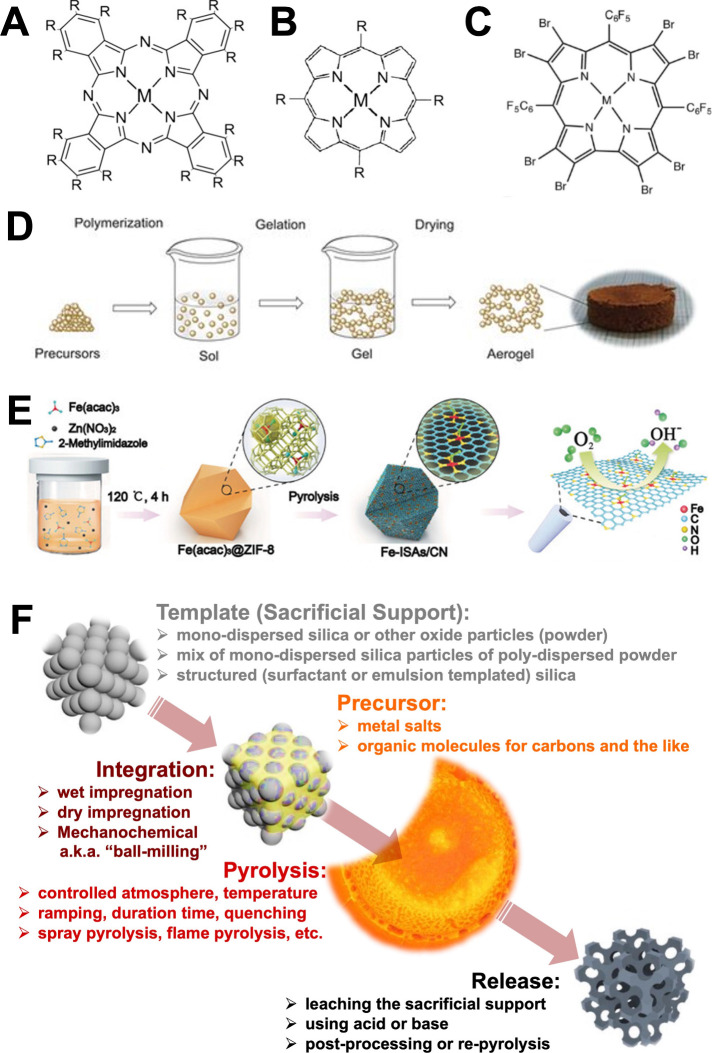
(A) Structure of the most commonly studied metal phthalocyanines. (B) Structure of the most commonly studied metal porphyrins. (C) Structure of the brominated metallocorroles [M­(tpfcBr8)]. (D) Schematic of aerogel synthetic preparation for ORR electrocatalysts. (E) Schematic procedure to fabricate Fe-based MOF-derived electrocatalysts. (F) Schematic of the sacrificial support method (SSM) synthetic procedure. (A) and (B) were arranged with permission from ref 16: Wiley, copyright 2016. (C) was arranged with permission from ref 19: Wiley, copyright 2015. (D) was arranged with permission from ref 25: Wiley, copyright 2021. (E) was rearranged with permission from ref 30: American Chemical Society, copyright 2017. (F) was rearranged with permission from ref 44: Elsevier, copyright 2020.

### First Synthetic Route: Azamacrocycles Pyrolyzed with a Conductive Porous Carbon Support

The most adopted azamacrocycles are phthalocyanine^8–14^ (Figure 1A), porphyrins^15–18^ (Figure 1B), corroles^19^ (Figure 1C), and others, where the TM is integrated within the structure. The mixture of TM-azamacrocycle and carbon substrate is subjected to pyrolysis at different temperatures and under different atmospheres (neutral or reducing). In general, temperatures lower than 400 °C do not allow robust integration of TM-N_*x*_ within the carbon structure. Temperatures higher than 600 °C led to the formation of metallic nanoparticles or oxides which are undesired; therefore, an acid washing to leach out these active surfaces is required.

### Second Synthetic Route: Azamacrocycle Polymer Pyrolyzed as a Covalent Framework (COF)

The linkage can occur through proper building blocks, linkage motifs, and diverse synthesis routes.^20,21^ However, the pyrolytic process is again used for increasing the electrical conductivity, enforcing graphitization while keeping the TM-N_*x*_ within the electrocatalyst structure.^22,23^ A successful example of efficient ORR electrocatalysts is based on the COF aerogels where the same or different TM-containing azamacrocycles are used and linked through condensation and then subjected to supercritical CO_2_ drying to create a defined 3D structure (Figure 1D).^24,25^ This structure undergoes a pyrolytic process in a controlled atmosphere and temperature environment to graphitize and enhance the conductivity. These electrocatalysts have high surface area, high tunable porosity, and high active site density.^26,27^ Also, in this case, eventual postpyrolysis treatment using acid washing can be pursued.

### Third Synthetic Route: Metal–Organic Frameworks (MOFs) Pyrolyzed to Obtain a Three-Dimensional Highly Porous Architecture

MOFs are crystalline materials consisting of metallic nodes coordinated with organic ligands.^28^ A huge variety of MOFs can be configured due to their tunable porosity and adjustable coordination chemistry. However, limited conductivity and structural stability are the main issues typically improved by subjecting them to high-temperature pyrolytic treatments.^29^ A typical fabrication route consists of pretreatments involving adjustments of the zeolitic imidazolate framework (ZIF) and proportion of the metallic species followed by a post-treatment that includes pyrolysis or calcination to form a robust structure.^30^ Final acidic treatments may also be included to etch out the course nanoparticles (Figure 1E). Pyrolysis converts the organic frameworks into the micromesoporous inorganic carbon matrix containing metallic species of interest in the forms of single-atom active sites, clusters, or nanoparticles.^31^ Single-atom metallic sites or metallic nanoparticles are formed during pyrolysis, depending on the pyrolysis temperature, improving the electrocatalytic ORR activities, whereas the layered micromesoporous networked structure enhances the mass transportation by shortening the diffusion distances and increasing the electode-electrolyte interface.^28^

### Fourth Synthetic Route: Metal Salt and Nitrogen-Rich Organic Precursor Pyrolyzed with a Templating Agent

The three compounds are mixed in optimized proportions and undergo pyrolysis under a controlled temperature and atmosphere. Soft^32–37^ and hard^38–43^ templating synthetic processes are used. For soft templating, urea, Zn, or other agents are removed during pyrolysis.^36^ For hard templating, the templating agent must be removed after the pyrolytic process.^44^ Many nitrogen-rich organic molecules and in parallel many metal salt precursors were investigated for this so-called sacrificial support method (SSM) reported in Figure 1F. Silica powders with diverse morphologies are the main hard-templating agent used.^45^ The removal of silica after the first pyrolysis creates a well-defined porosity. In the original SSM approach, silica is used as a templating agent mixed with metal salt and a N-rich organic molecule. After a first pyrolysis step, the silica is etched out using hydrofluoric acid (HF)^46–48^ or other alternatives to HF but capable of generating HF in situ,^49^ or recently, Teflon was used during pyrolysis to operate as an etching agent during the pyrolytic process.^50,51^ The obtained TM-N_*x*_-C ORR electrocatalyst undergoes a second pyrolysis in a hydrogen or ammonia rich atmosphere to improve graphitization and clean out the TM-N_*x*_ active sites.^52,53^ The US-based company Pajarito Powder has adopted this synthetic pathway to fabricate at a large scale and commercialize PGM-free ORR electrocatalysts.^54^

## Description of the Oxygen Reduction Reaction Active Sites

State-of-the-art and benchmark PGM-free electrocatalysts are synthesized by using high-temperature processes. The diversity of synthetic routes, precursor used, and pyrolysis conditions create different (TM)-N_*x*_C moieties incorporated in diverse carbon matrixes. The high-temperature process is central in (i) forming the functional groups containing (TM)-N_*x*_C, enhancing the number of desired active sites, (ii) integrating the active sites within the graphitic backbone, but also (iii) enhancing the degree of graphitization of the carbon support.^55^

The nitrogen moieties can be located on the edges or in-plane defects. Some of them present good activity for the oxygen reduction reaction and are therefore designated as “active sites”. Metal nanoparticles are also formed in the form of metal carbides, nitrides, or oxides. These nanoparticles are, in general, undesirable for PEMFC catalysts and are avoided or removed via leaching. A general representation of the nitrogen-containing and iron-containing species for a Fe-containing (TM)-NC is shown in Figure 2A,B.^55^ The nature of these sites has been extensively studied using *ex situ* and *in situ* X-ray photoelectron spectroscopy (XPS)^56–60^ and XAS,^61–68^ and by combining electrochemical and XPS measurements with density functional theory calculations.^69^

**Figure 2:**
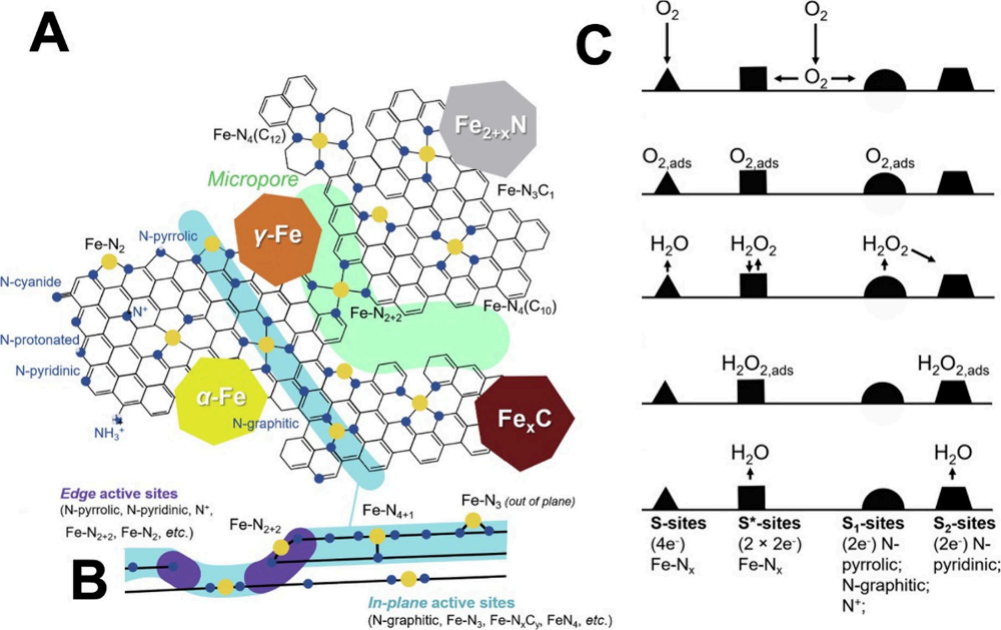
(A) The plurality of active sites observed in Fe-N_*x*_-C electrocatalysts. (B) In-plane and edge defects in Fe-N_*x*_-C electrocatalysts. (C) Oxygen reduction reaction mechanism on the different active sites (S, S*, S1, and S2) commonly observed in Fe-N_*x*_-C electrocatalysts. (A), (B), and (C) were rearranged with permission from ref 55: Elsevier, copyright 2020.

The moieties that are active sites for ORR have been determined to have the transition metal coordinated with nitrogen in a pyridinic environment, forming a TM-N_*x*_ site with TM as Mn, Fe, Co, Ni, or Cu and *x* equal to 2, 3, or 4. Fe is by far the most used metal, as it demonstrates the highest activity thanks to a high redox potential of the couple Fe^2+^/Fe^3+^ and a higher affinity for molecular oxygen. Fe-N_*x*_ can perform the ORR in a direct 4e^–^ transfer mechanism (S-site) or can produce peroxide in a 2e^–^ transfer. Via a second 2e^–^ transfer, the peroxide can be reduced to water (2 + 2e^–^ pathway) within the same site (then called S*-site) or on another peroxide-reducing site. Pyrrolic and graphitic nitrogen defects of the graphitic structures without TM are designated as S1 sites because they mostly reduce oxygen to peroxide. However, pyridinic nitrogen defects are suspected to be a secondary metal-free active site able to further reduce peroxide (S2-sites).^58^ In Figure 2C, the different active sites and their implications within the ORR are clearly represented. The activity for the different reactions and pathways can however vary with the electrolyte considered.^45^

During the heat treatment, the morphological features of the electrocatalysts like the primary particle size, pore size distribution, tortuosity, and (active and electrochemical) surface area depend on the synthesis route used, formed during the heat-treatment step.^55^ The morphological features are critical as they guarantee the accessibility of the reactants to the active sites and product removal from the surface.^55^

The pyrolysis temperature, dwell at high temperature, and heating/cooling rate can affect both surface chemistry and morphology.^70–72^ The atmosphere used during the different heat-treatment steps, neutral (N_2_ or Ar) or reductive (NH_3_ or H_2_), has a tremendous effect on the final catalyst’s properties.^44,73,74^

## Elucidating Chemical and Morphological Transformation during Pyrolysis

Up to a few years ago, pyrolytic processes to synthesize ORR Fe-N_*x*_-C electrocatalysts were considered as a “black box” and the correlations between surface chemistry and morphology and pyrolytic conditions were inferred only at the end of the process. The exact mechanism of formation of the Fe-N_*x*_-C active sites during the pyrolysis step was unknown, and this hindered rational synthesis design. This knowledge gap forced most of the approaches to be empirical and many open questions were raised within the scientific community.^70^

Only in the last 5 years has some light has been shed on the pyrolysis step using in situ techniques, often supported by synchrotron techniques. The active site formation and variations of morphological features were tracked along the process.

The first breakthrough related to understanding the pyrolysis process was introduced recently in 2019, when Li et al.^75^ directly observed the evolution pathway from the precursors to single-atom Fe_1_(II)-N_4_ORR-active electrocatalyst via in situ X-ray absorption spectroscopy (XAS). They monitored two different synthetic routes: the first using iron chloride (FeCl_2_·4H_2_O) and a heat-treated N-doped carbon matrix (N-C) and the second using iron­(II) acetate (FeAc_2_), 1,10-phenanthroline monohydrate, and ZIF-8 (a Zn-based MOF).^75^

In situ XAS conducted during pyrolysis showed that the Fe precursor transforms to Fe oxides below the pyrolytic temperature of 300 °C. Further increase in temperature (below 600 °C) leads to a crystal-to-melt-like transformation to tetrahedral Fe_1_(II)-O_4_. With the further increase in pyrolysis temperature (up to 1000 °C), XANES spectra showed the formation of in-plane Fe_1_(II)-N_4_ resembling the spectra of Fe­(II) phthalocyanine (FePc)^75^ (Figure 3A).

**Figure 3:**
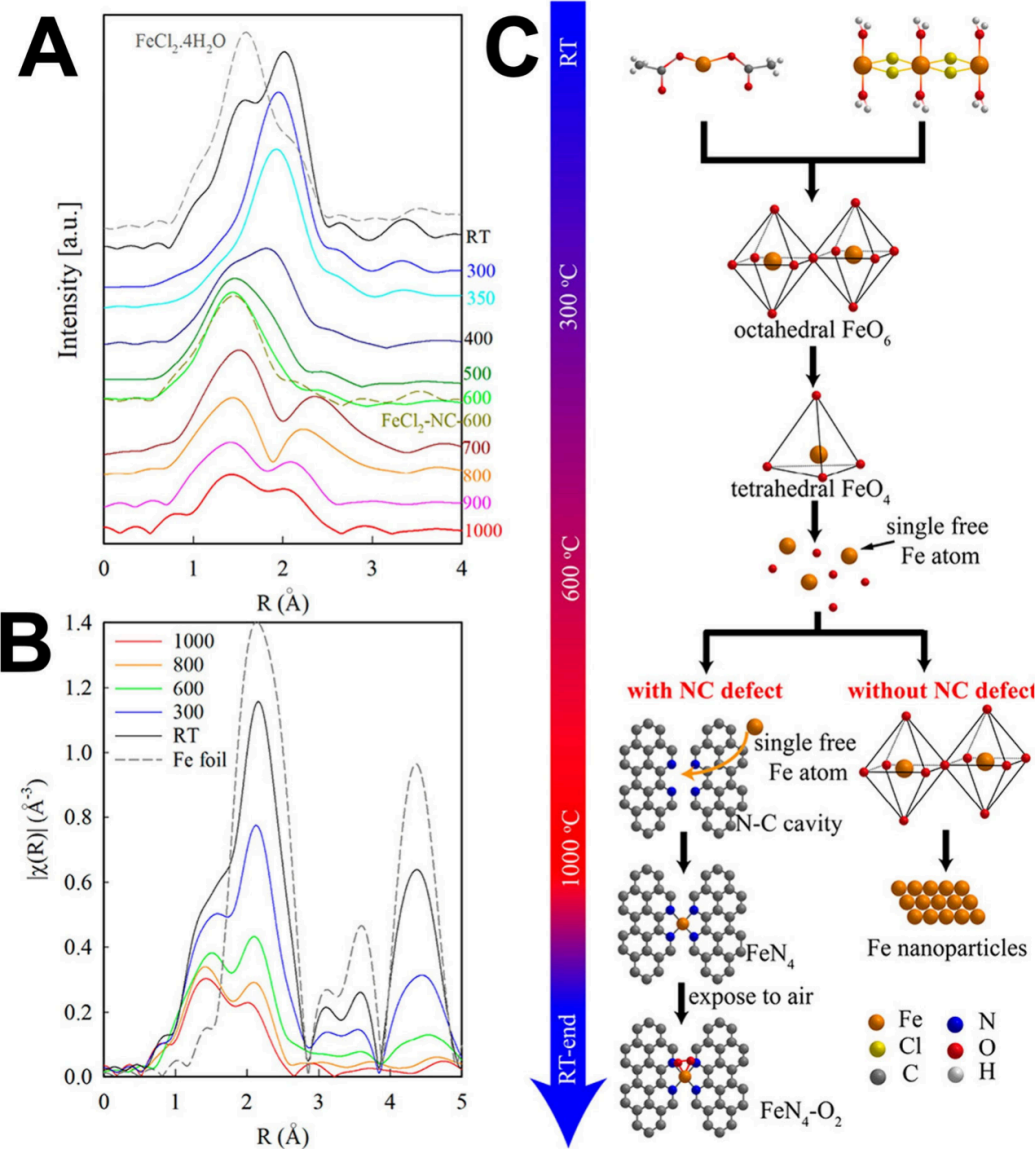
FT-EXAFS spectra of FeCl_2_·4H_2_O mixed with silica collected with (A) increasing temperature from RT to 1000 °C and (B) cooling down from 1000 °C to RT. (C) Thermal evolution of iron compounds during the pyrolytic process. The two diverging cases with the presence or absence of N-C defects are also presented. Rearranged with permission from ref 75.

Interestingly, the transformation continues during the cooling down phase to room temperature, indicating that the transformation is an irreversible thermal process (Figure 3B). After the exposure to air, the XANES spectrum of the Fe-N_*x*_-C electrocatalyst shifted positively, indicating the surface Fe_1_(II)-N_4_ oxidation via adsorption of an O_2_ or OH ligand, and the formation of Fe_1_(III)-N_4_-O_2_/OH sites. A similar thermal transformation pathway was observed in the in situ XAS experiment on the MOF-based mixture: Fe precursor → Fe oxides (octahedral Fe-O_6_) → tetrahedral Fe_1_(II)-O_4_ → Fe_1_ → Fe_1_(II)-N_4_.^75^

This trend is shown in Figure 3C, where transformation mechanisms are reported along with the pyrolysis temperature focusing on the Fe part.

This result indicates that any precursor containing Fe, N, and C that undergoes similar pyrolysis will likely form Fe_1_(II)-N_4_. The mechanism of transformation of tetrahedral Fe_1_(II)-O_4_ to in-plane Fe_1_(II)-N_4_ could not be fully resolved but the authors hypothesized the transport of the Fe_1_ through the gas phase to the N_4_-C defect with the formation of the desired Fe_1_(II)-N_4_.^75^

The question of morphological evolution cannot be resolved with XAS measurements alone. This knowledge is particularly needed for a better understanding of the mechanism of creation and evolution of the active sites during the pyrolytic process.

An initial answer to this important question was given by Huang et al., who monitored the morphological development and the chemical transformation of an electrocatalyst synthesized through the sacrificial support method (SSM).^76^ This work focused on the first pyrolytic process, to mimic closely the actual protocol, where two temperature ramps were used to reach 975 °C for 1 h. In this synthesis a nitrogen-containing charge transfer organic salt was used as a nitrogen–carbon precursor, iron nitrate as the iron source, and amorphous silica powder as the hard templating agent. A combination of *in situ* light synchrotron and lab-based diagnostic techniques including micro and nano X-ray computed tomography (X-ray CT), X-ray diffraction (XRD), and scanning transmission electron microscopy (STEM) were used to evaluate the variation of the morphological features of the N-C backbone matrix across a temperature–time trajectory occurring during the first pyrolysis.^76^ Techniques utilized in this work are summarized in Figure 4A.

**Figure 4:**
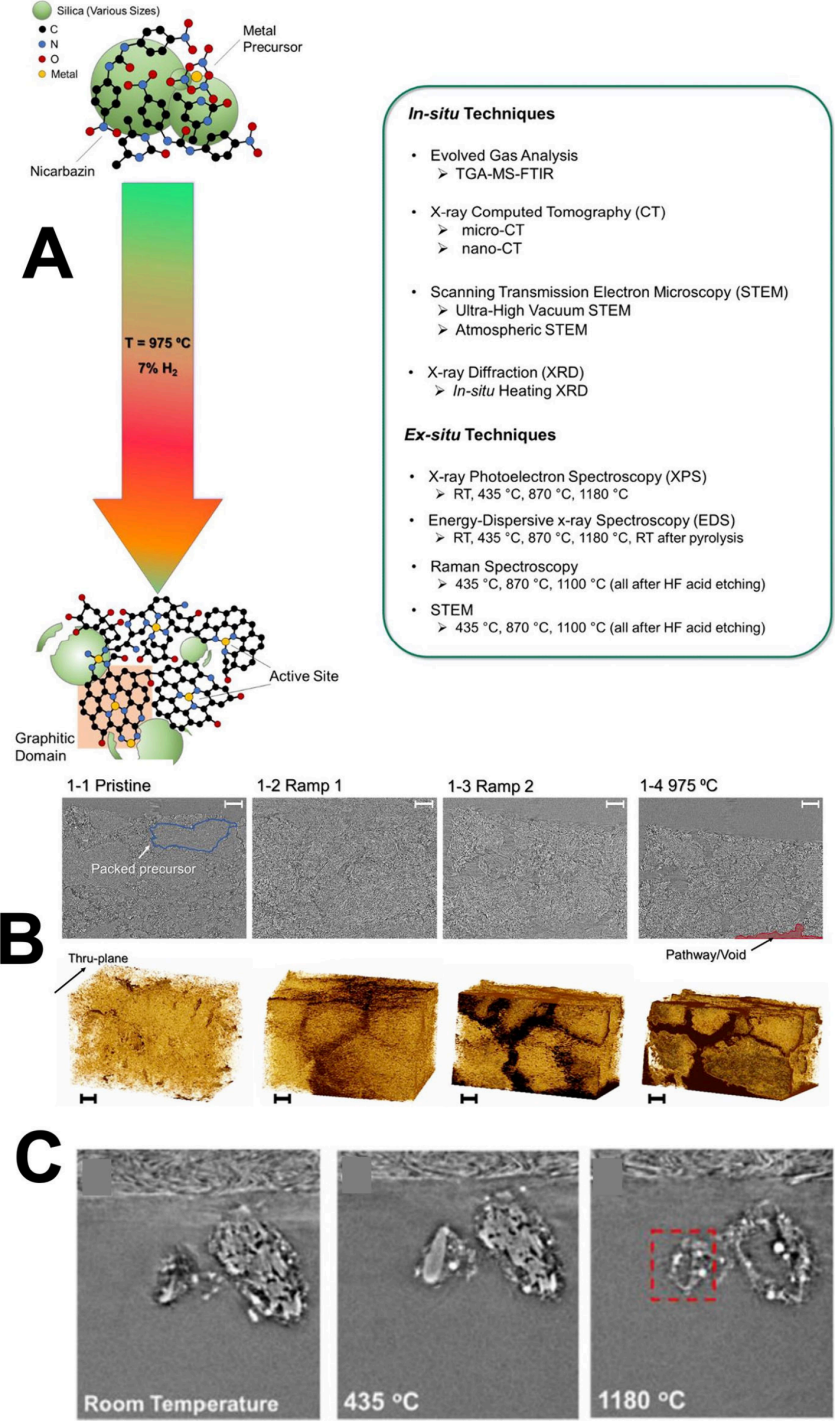
(A) List of the techniques used in the study. (B) cross-section images representative of the sample subject to pyrolysis and 3D structures created based on the image stacks using nano X-ray CT. (C) Representative X-ray cross-section tomographs during the temperature ramping using *in situ* nano-CT analysis (pyrolysis atmosphere 5% H_2_ and 95% N_2_). Rearranged with permission from ref 76: Elsevier, copyright 2021.

X-ray CT was used to observe and identify the morphological evolution of the precursors at micro- and nano-scales, evaluating the material loss, particle size change, and porosity change during pyrolysis steps in a furnace with X-ray transparent windows. During the temperature ramp, an increase of the porosity of the mixture was measured due to water evaporation, iron nitrate decomposition, and the nicarbazin melting and decomposition. The porosity then decreased once the temperature was held at high values (975 °C), as the precursor settles. Micro X-ray CT does not have sufficient resolution to observe morphological transformations at particle scale and only large-scale (>1 um) transformations can be observed. The gas evolution leads to an increase of the macro-scale porosity. This macro-scale porosity might not be preserved after mixing the electrocatalyst or processing it in another way (such as ball milling) (Figure 4B). Through nano X-ray CT with a resolution of 30 nm and field-of-view of about 80 μm, the authors observed single particle transformation during pyrolysis. The amorphous carbon formed was decomposed at temperatures above 870 °C, forming graphitic shells visible at 1180 °C (Figure 4C). Some particle shrinkage was observed too. The bright particles observed are Fe or Fe-carbide nanoparticles that form at higher temperatures.^76^

Morphological transformations at the nano-scale were also investigated using environmental STEM. It showed that the organic precursor decomposed (melting and evaporation), while the silica remained unchanged during the heat treatment and the iron metallic clusters disappeared between 710 and 870 °C, becoming atomically dispersed.^76^

The X-ray radiography images showed that nicarbazin liquified around 263 °C, filled the voids, and covered the silica templating, occupying the entire volume while gas bubbles were formed and evacuated.^76^

The authors divided the pyrolytic process into three different parts: (1) at temperatures below 435 °C, the N-C precursor used, nicarbazin, a charge transfer salt, melts and decomposes; (2) at temperatures between 435 and 870 °C, amorphous carbon domains are formed and metallic iron nanoclusters disperse in the carbon matrix; (3) at above 870 °C, graphitization of the carbon, formation of Fe-N_*x*_ moieties, and further aggregation of the single transition metal atoms into nanoparticles occur.^76^

In general, Fe-N_*x*_-C electrocatalysts need to undergo secondary treatments (e.g., acid washing) to be efficient and stable toward the ORR. Particularly, PGM-free electrocatalysts synthesized using SSM need to undergo silica templating etching using hydrofluoric acid (HF) and create the desired porosity.^49^ After the etching, a second pyrolysis in a reducing atmosphere is usually pursued showing improved electrochemical performance toward the ORR and stability during operations.^44^

Regarding this matter, very recently, Chen et al. focused their attention on the second pyrolysis of a Fe-N_*x*_-C electrocatalyst synthesized using SSM (nicarbazin as N-C precursor, Fe-nitrate as Fe precursor and silica) after HF treatment to remove the silica templating agent.^77^ The second pyrolysis after HF etching was followed by different characterization methods including *in situ* heating XPS, STEM, energy-dispersive X-ray spectroscopy (EDS), electron energy loss spectroscopy (EELS), XRD, and X-ray CT.^77^ Through in situ heating XPS, the rearrangement and transformation of the diverse and multitudinous N moieties were resolved.

Particularly, the repyrolysis process produced a more balanced N-C structure. A small decreasing fraction of edge pyridinic nitrogen sites (prone to protonation and hydrogenation), a stabilization of metal-N, an important decrease in surface and bulk N–H sites, and an increase in graphitic nitrogen were observed. Interesting results were obtained from *in situ* UHV heating STEM, where it was noticed that the agglomeration of metallic clusters disintegrates with the increase in temperature, diffusing into the carbon structure and leading to smaller agglomerates or atomic dispersion (Figure 5). Remarkably, diffusion became faster at higher temperatures. It was also evidenced that when the temperature decreased, the metal remained atomically dispersed. EELS and EDX have shown atomically dispersed diffusion and distribution within the electrocatalyst subjected to repyrolysis.^77^ *In situ* XRD did not show any formation or decomposition of large crystals for Fe-N_*x*_-C electrocatalysts. Even X-ray CT did not show major changes in the morphology during the second pyrolysis.^77^

**Figure 5:**
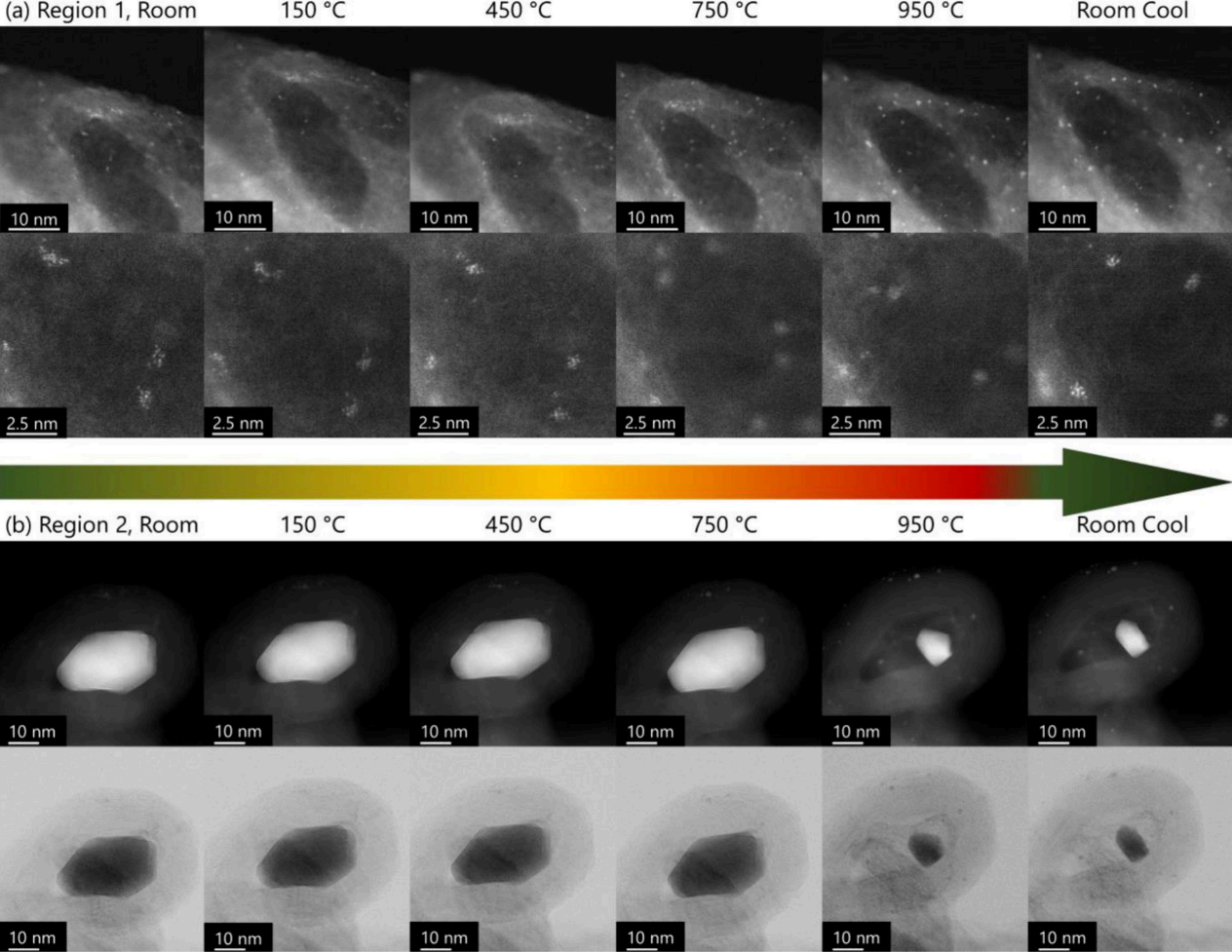
STEM images of *in situ* UHV heating of Fe-N_*x*_-C electrocatalysts. (top) The cluster/single atom region. (bottom) The nanoparticle/carbide region. The bottom row of both corresponds to bright field version of the top row. Rearranged with permission from ref 77: Elsevier, copyright 2022.

The same authors have also investigated the pyrolysis processes leading to the transformation of a metal–organic framework (MOF) into the TM-N_*x*_-C electrocatalyst. Two MOFs possessing the same sodalite topology were considered and particularly ZIF-8 and ZIF-67 were investigated.^78^ Their chemical and structural evolution and transformation under high-temperature treatment were evaluated using diverse *in situ* and *ex situ* techniques. Initial thermogravimetric analyses have revealed the temperatures of melting and decomposition of ZIF-8 and ZIF-67, both being purely crystalline under an XRD initial evaluation. Pyrolysis changes significantly the morphology of the MOF when it was transformed to M-N_*x*_-C materials.^78^

Concerning ZIF-8, during pyrolysis, the shape remained the same, but a a size shrinkage of up to 45% at 850 °C was noticed. The crystal structure of ZIF-8 underwent decomposition or amorphization at temperatures above 520 °C.^78^

Concerning ZIF-67, metallic agglomeration was assessed during the pyrolytic process. The shape of this MOF collapsed, and graphitization took place, supported by the transition metal. Raman spectroscopy supported this statement too, with the MOF that was fully transformed to carbonaceous materials at 650 °C.^78^

In general, the most pronounced difference between the pyrolyzed product sof ZIF-8 and ZIF-67 was the formation of the metallic particles in the latter MOF. Moreover, MOFs have high structural porosity that benefits the electrocatalytic activity; however, a pyrolytic process is needed to enhance graphitization and in turn electrical conductivity. In general, the presence of a transition metal within the MOF leads to the formation of metal aggregates and can favor the graphitization and consequent collapse of the structure. ZIF-8 is a promising MOF as it contains Zn as a transition metal that evaporates during pyrolysis; therefore, no graphitization is enhanced and the structure is maintained during pyrolysis.^78^

Very recently, the understanding of the active sites formation was enhanced with direct observation of the evolution of Fe-N_*x*_-C starting from azamacrocycles containing iron (Fe-phthalocyanine) with the variation in the pyrolysis temperature through in situ XAS (XANES and EXAFS) during the pyrolytic process.^79^

In fact, Muhyuddin et al. evaluated the formation of Fe-N_*x*_-C active sites starting from a high surface area and highly graphitized carbon black (Ketjen Black-600) mixed with iron­(II) phthalocyanine (FePc). The source of carbon was given by the Ketjen Black-600 and FePc already exists in the desired active site structure. A detailed investigation was conducted using a microtome to explore the effect of the pyrolysis temperature and atmosphere during the pyrolytic process on the active sites’ formation and development.^79^ Differently from the synthetic processes described before where an acid washing is needed after the first pyrolysis,^44^ this proposed pyrolytic process does not utilize a final acid washing. Therefore, *in situ* XAS (XANES and EXAFS) during pyrolysis was used to track the variation of the initial Fe-N_*x*_ active sites of the FePc. XANES data related to *in situ* and *ex* *situ* pyrolysis and the effect of the pyrolysis atmosphere on the Fe speciation is reported in Figure 6.

**Figure 6:**
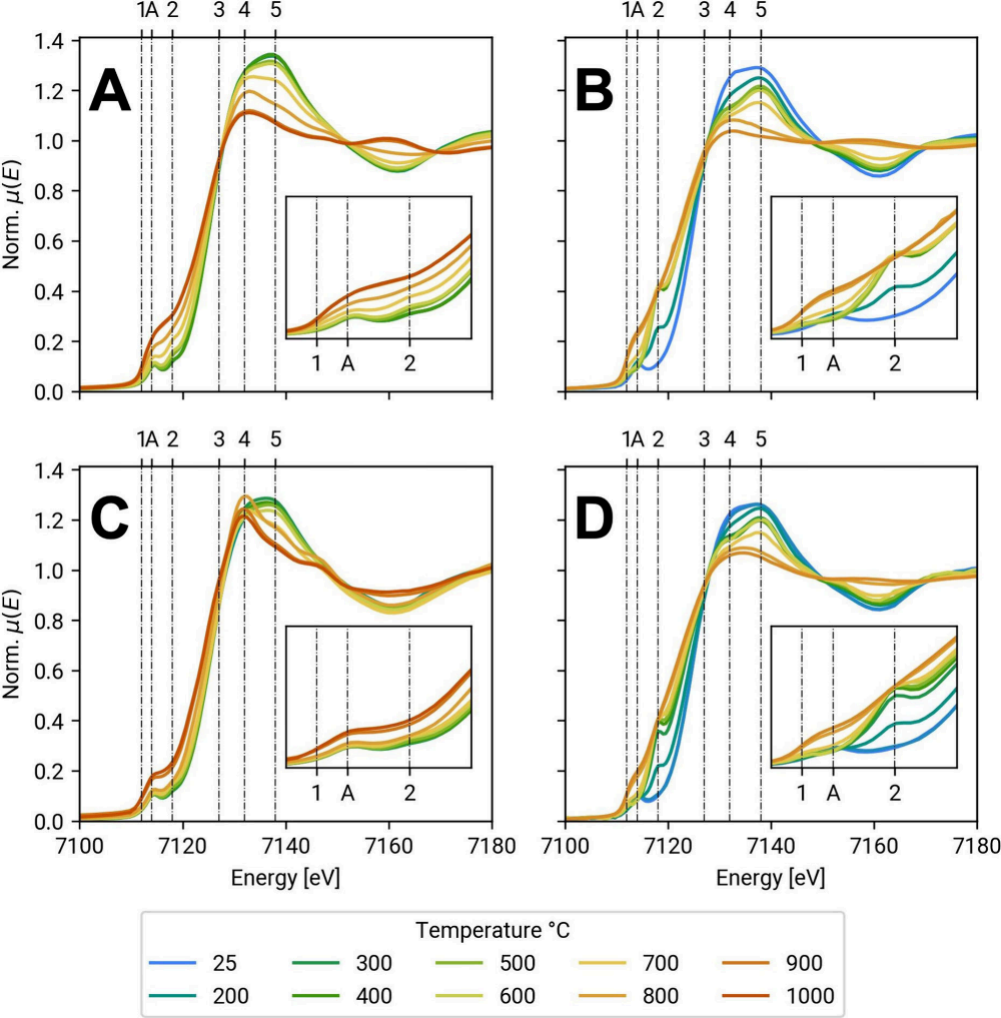
XANES profiles of FePc: (A) spectra collected *ex situ* in Ar/H_2_ atmosphere, (B) spectra collected *in situ* in Ar/H_2_ atmosphere, (C) spectra collected *ex situ* in Ar atmosphere, and (D) spectra collected *in situ* in Ar atmosphere. Rrearranged from ref 79: Elsevier, under Creative Commons license CC-BY 4.0.

The data obtained showed that atomically dispersed active sites, type Fe-N_*x*_, were maintained up to a 600 °C pyrolytic temperature. At temperatures higher than 600 °C, aggregation took place and nanoparticles and oxides were detected. This mechanism was also supported by *ex situ* XRD. The exposure of the electrocatalyst to air leads to the interaction of the Fe with O_2_ molecules and the formation of Fe_1_(III)-N_4_-O_2_/OH sites. Structure–property analysis was carried out including a series of *ex situ* measurements such as HRTEM, XPS, Raman, etc. The best-performing electrocatalyst was the one pyrolyzed at 600 °C, indicating a clear link between the desired active sites and the ORR performance.

Similarly, Ni-N_*x*_-C active sites were evaluated during the pyrolytic process starting from high surface area and highly graphitized carbon black (Ketjen Black-600) mixed with nickel­(II) phthalocyanine (NiPc). Similarly to the situation described for FePc/C, NiPc/C remained atomically dispersed until 600 °C with the structure Ni-N_*x*_.^80^ Above 600 °C, Ni-N_*x*_ and Ni nanoparticles coexisted, with the latter increasing in size with temperature. Interestingly, when exposed to air, their XAS (XANES and EXAFS) did not vary, indicating a low or no interaction of Ni with the oxygen molecule. NiPc/C pyrolyzed between 200 and 1000 °C did not have good performance for the ORR compared to FePc/C and this is due to the interaction between O_2_ and Ni and the reaction mechanism which is more toward peroxide production (2e^–^ transfer). NiPc/C pyrolyzed at temperature above 600 °C, where Ni nanoparticles were present, showed improved performance for the hydrogen evolution reaction (HER).^80^

## Conclusions and Perspectives

In the past few years, important achievements have been reached to decipher and understand the processes occurring within the pyrolytic process to synthesize PGM-free ORR electrocatalysts. These novel works unravel the processes occurring within pyrolysis on the surface chemistry and morphology. Related to surface chemistry, *in situ* measurements mainly focus on the formation of the active site Fe-N_*x*_ starting from diverse precursors using XAS (XANES+EXAFS). The iron precursor is transformed into oxides and then with increasing temperature into atomically dispersed iron that through gaseous transport migrates to N-C defects over the graphitic structure, forming Fe coordinated with nitrogen on the graphitic backbone. Also, the carbon–nitrogen precursors are carbonized with temperature, creating an amorphous-graphitic structure over time. Interestingly, a second pyrolysis also led to the rearrangement of N functionalization and spreading of the iron with the formation and redistribution of Fe-N_*x*_-C active sites on the electrocatalyst surface. In the case of utilization of precursors already possessing the Fe-N_*x*_ initial chemical structure, as for example FePc or NiPc, an increase in temperature led to the formation of nanoparticles due to the coalescence of the single metallic atoms above 600 °C.

*In situ* X-ray CT unravels the decomposition and evaporation of the carbon–nitrogen precursor (in this case nicarbazin) and the creation of the porosity due to bubble expulsion as well as the melting of the precursors. Moreover, *in situ* X-ray CT was also able to check the structural integrity of different MOFs subjected to pyrolysis.

These recent works have made an important contribution to understanding the processes occurring within the pyrolytic process that up to now were completely unknown. Direct visualization of pyrolytic processes with instruments capable of directly following chemical and morphological changes is a complete breakthrough. These routes open up diverse new pathways to be pursued not only for understanding synthetic processes using pyrolysis but also expanded to other categories of materials where surface chemistry is crucial (e.g., electrocatalysis or heterogeneous or homogeneous catalysis) or where surface morphology is crucial (e.g., biochar production). We are certain that these achievements are essentially required by the scientific community to answer questions, unknown up to now, related to high-temperature treatment in a controlled atmosphere and temperature. We are confident and hope that these techniques can be successfully translated and useful to push to the next generation the development and synthetic processes of many other materials for electrochemistry, energy production, environmental processes, and other applications that can further contribute to the decarbonization process currently taking place.
